# Impact of Acute Kidney Injury on Hospitalization Outcomes in Patients with Chronic Kidney Disease Undergoing Cardiac Procedures with Cardiopulmonary Bypass in the United States

**DOI:** 10.36469/001c.163558

**Published:** 2026-08-14

**Authors:** Nicolas Pope, Abby Greenblatt, Thainá Jehá, Juliana Meyers, Christine M. Solinsky, Rakesh C. Arora, Yan Wang

**Affiliations:** 1 Medical University of South Carolina, Charleston, South Carolina; 2 Real World Data & Analytics, RTI Health Solutions, Research Triangle Park, North Carolina; 3 Alexion, AstraZeneca Rare Disease, Boston, Massachusetts; 4 Harrington Heart & Vascular Institute, University Hospitals, Cleveland, Ohio

**Keywords:** cost-effectiveness analysis, lung transplant, chronic lung allograft dysfunction, decision model, extracorporeal photopheresis

## Abstract

**Background:**

Acute kidney injury (AKI) is a common complication after cardiac surgery and is associated with more infections, prolonged recovery, longer hospital stays, increased readmission rates, greater costs, and higher mortality. Patients with pre-existing chronic kidney disease (CKD) are at particularly high risk, with this risk increasing as renal function worsens. Damage caused by AKI may be irreversible, despite a return to baseline GFR, compounding stress on remaining nephrons. Understanding risk in these populations may ultimately help to improve prevention and outcomes.

**Objective:**

Evaluate the impact of AKI on outcomes and healthcare costs among adults with CKD undergoing cardiac surgery with cardiopulmonary bypass (CPB).

**Methods:**

Multi-institutional, retrospective, observational study using the Healthcare Cost and Utilization Project Nationwide Readmissions Database, 2016-2020 datasets. The total adult cardiac surgery population were those with CKD who underwent cardiac surgery requiring CPB (reporting incidence); from which a subpopulation of adults with severe CKD (stage 3 or 4; based on ICD-10-CM codes during index hospitalization) was assessed (impact of AKI). Patients were categorized as follows, based on AKI status during index hospitalization: no AKI, AKI, or AKI requiring dialysis (a subpopulation of AKI).

**Results:**

AKI incidence during initial hospitalization was 55.9% and 77.3% in patients with CKD stage 3 and 4, respectively. AKI requiring dialysis occurred in 3.4% and 14.5% of CKD 3 and 4 patients, respectively. One-third of all AKI cases and half of AKI requiring dialysis cases occurred in patients with CKD stage 3 or 4. In patients with CKD stage 3 or 4, multivariate analysis showed AKI was associated with increased inpatient days, increased risk of 180-day readmission, higher hospitalization costs, and greater odds of in-hospital mortality. Patients experienced the greatest burden when AKI required dialysis.

**Conclusions:**

Incidence of AKI is higher in patients with preexisting CKD stage 3 or 4 vs 1 or 2 and those without CKD. The burden of AKI is elevated in patients with CKD undergoing cardiac surgery with CPB; targeted interventions are needed to prevent poor outcomes.

## BACKGROUND

Acute kidney injury (AKI) is a frequent complication following cardiac surgery, particularly in patients with impaired baseline kidney function. AKI manifests as a sudden decline in kidney function, typically indicated by elevated serum creatinine (sCr) or reduced urine output.^1–5^ A large prospective, observational study using pooled incidence rates from 30 countries reported that 25.9% of patients undergoing cardiac surgery developed post-surgery AKI.^6–8^ Postoperative AKI is associated with poor outcomes, including prolonged recovery times and high rates of complications, infections, and mortality.^9^ These poor outcomes often lead to an increased length of stay (LOS), heightened risk of readmission, and elevated healthcare costs.^1–3^

Patients with chronic kidney disease (CKD) who undergo cardiac surgery with cardiopulmonary bypass (CPB) are at a significantly greater risk of developing AKI during hospitalization than those without CKD.^10–12^ This risk increases with worsening kidney function. Diagnosis of both CKD and AKI in adults is routinely made using estimated glomerular filtration rate (eGFR). However, decreases in eGFR are a late and imperfect marker, as milder kidney dysfunction can be masked by the renal functional reserve.^13^ Consequently, eGFR and urinalysis might not be sensitive enough to detect mild yet clinically significant kidney damage.^14^ This is an important consideration, as it has been suggested that even ‘subclinical’ AKI negatively impacts outcomes post-cardiac surgery.^15,16^ Furthermore, the damage caused by AKI may be irreversible, despite a return to normal baseline GFR; this can result in progression of kidney disease, due to increased stress on remaining nephrons.^17^ Therefore, quantifying the preoperative risk of cardiac surgery-associated AKI may provide important information to improve both preventative practice and patient outcomes.

Using data from the Healthcare Cost and Utilization Project (HCUP) Nationwide Readmissions Database (NRD), this study first evaluated the incidence of AKI in adult patients with any stage CKD (no CKD to CKD stage 4) undergoing specific cardiac procedures with CPB in the United States (US) (incidence population). Subsequently, within a subpopulation of this incidence population, the impact of AKI on hospitalization outcomes (including LOS, readmission rates, and mortality) and healthcare costs among adult patients with severe CKD (CKD stages 3 and 4) was assessed. The study also compared demographics, hospital characteristics, clinical characteristics, and outcomes among patients within this subpopulation depending on whether they developed AKI.

## METHODS

This retrospective, observational study utilized deidentified data from the HCUP NRD. The NRD is derived from the HCUP State Inpatient Databases, on average accounting for 60.3% of the total US population and 59.0% of all US hospitalizations annually. The study analyzed data from 2016 to 2020 (inclusive). For more information see **Supplementary Methods**.

As this was a retrospective observational study–with no direct access to patient data or interventions–no informed consent was solicited and RTI International’s Institutional Review Board determined that this study was not research involving human subjects. Deidentified data ensured patient privacy was protected. All relevant laws and institutional guidelines were adhered to throughout this study.

All reported conditions and comorbidities were identified using the International Classification of Diseases, Tenth Revision, Clinical Modification (ICD-10-CM) system; all procedures were classified using the ICD-10 Procedure Coding system (ICD-10-PCS). A table of diagnosis and procedure codes and scoring criteria can be found in **Supplementary Table S1**.

### Incidence of AKI in the Total Adult Cardiac Surgery Population

**Figure 1.**
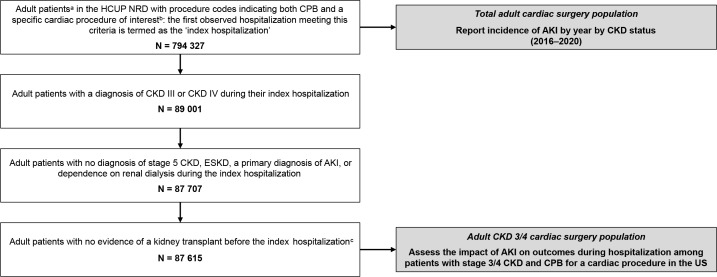
Patient Selection and Cohort Criteria Abbreviations: AKI, acute kidney injury; CKD, chronic kidney disease; CPB, cardiopulmonary bypass; ESKD, end-stage kidney disease; HCUP, Healthcare Cost and Utilization Project; NRD, Nationwide Readmissions Database; US, United States. ^a^Aged ≥18 years at the time of admission to the index hospitalization. ^b^Either the CPB or the cardiac procedure was coded as the primary procedure. ^c^Patients had no diagnosis indicating a previous kidney transplant and no evidence of a transplant procedure.

The incidence of AKI within the total study population was reported. The total study population comprised all adult patients within the HCUP NRD undergoing a cardiac procedure of interest during the analysis period (**Figure 1**).

To determine AKI incidence, data from adult patients who underwent multivessel coronary artery bypass graft (CABG), valve replacement/repair, or combined CABG and valve replacement/repair with CPB were analyzed. The incidence of AKI was reported in subgroups based on CKD status (no CKD, CKD stages 1-4) as determined via ICD-10-CM codes. Additionally, the incidence of AKI and AKI requiring dialysis (a subgroup of AKI) was reported overall and within each CKD stage, categorized by age and sex, and tracked longitudinally by hospitalization year.

AKI incidence was calculated as the weighted number of hospitalizations with AKI divided by the weighted total number of hospitalizations, ×1000; incidence is reported as percentages. Discharge-level weightings are provided by HCUP; full descriptions of data weighting can be found at the HCUP website (https://hcup-us.ahrq.gov/db/nation/nrd/Introduction_NRD_2010-2018.jsp).^18^

### Impact of AKI on Outcomes in the Adult CKD 3/4 Cardiac Surgery Population

To assess the impact of AKI in patients with CKD stage 3 or 4 (CKD 3/4), patients with CKD stage 1 or 2, or without CKD during the index hospitalization were excluded from all impact of AKI outcomes analysis. Patients were also excluded from the impact analysis if they received a diagnosis of CKD stage 5, end-stage kidney disease (ESKD), a primary diagnosis of AKI, or had received a prior kidney transplant (**Figure 1**).

Two mutually exclusive cohorts were analyzed among adults within the CKD 3/4 cardiac surgery population: patients who developed AKI (“AKI” cohort), and those without AKI (“no AKI” cohort) during index hospitalization (**Figure 1**). A subset of patients with AKI who required dialysis were also assessed (“AKI requiring dialysis” cohort). Index hospitalization was defined as the first observed hospitalization that met study selection criteria (**Figure 1**).

A range of demographic and clinical characteristics were reported (**Tables 1 and 2**, **Supplementary Tables S2-S6**). Procedures and conditions of interest during index hospitalization included intra-aortic balloon pump, cardiogenic shock, and hypervolemia. Key study outcomes included LOS, costs (US dollars), and mortality for index hospitalization; clinical outcomes of interest (postoperative cardiogenic shock, modified failure to rescue, surgical site infection); and readmissions (30, 60, and 180 days following index discharge). All costs were converted from hospital charges and expressed in 2023 US dollars, using the Consumer Price Index (medical care component) to adjust for inflation. Modified failure to rescue was defined as mortality during a hospitalization where a patient had one or more of the following: prolonged mechanical ventilation, stroke, and/or CKD 5/ESKD.

**Table 1.** Demographic Characteristics of Hospitalizations for Adults with CKD 3/4 Who Underwent Multivessel CABG, Valve Replacement/Repair, or CABG and Valve Replacement/Repair with CPB

**Characteristic**	**No AKI (N = 36 948)**	**AKI (N = 50 667)**	**AKI Requiring Dialysis^a^ (N = 3485)**	***P* Value (No AKI vs AKI)**	***P* Value (No AKI vs AKI Requiring Dialysis)**
Age, years, % (n)					
18-34	0.2 (70)	0.4 (189)	–^b^	.0034	.0575
35-44	0.8 (285)	1.3 (674)	2.0 (71)	<.0001	<.0001
45-54	4.5 (1661)	5.5 (2811)	6.7 (234)	<.0001	.0001
55-64	17.7 (6528)	18.7 (9493)	18.3 (639)	.0085	.5039
65-74	41.0 (15 143)	38.8 (19 657)	36.1 (1258)	<.0001	.0001
75-84	32.5 (12 002)	31.7 (16 037)	32.8 (1145)	.0910	.7804
≥85	3.4 (1259)	3.6 (1806)	3.6 (124)	.4150	.7717
<65	23.1 (8544)	26.0 (13 167)	27.5 (959)	<.0001	.0001
≥65	76.9 (28 404)	74.0 (37 499)	72.5 (2527)	<.0001	.0001
Mean (SE)	70.5 (0.1)	69.8 (0.1)	69.5 (0.3)	<.0001	<.0001
Median	71	71	71		
Range (Q1, Q3)	65, 76	64, 76	63, 77		
Sex, % (n)					
Male	71.0 (26 239)	72.9 (36 957)	67.5 (2353)	.0001	.0042
Female	29.0 (10 709)	27.1 (13 710)	32.5 (1132)	.0001	.0042
Insurance payer type, n (%)					
Medicare	73.6 (27 177)	71.4 (36 171)	72.6 (2530)	<.0001	.4351
Medicaid	4.3 (1599)	6.4 (3251)	7.4 (257)	<.0001	<.0001
Private insurance	18.2 (6728)	17.4 (8825)	15.0 (523)	.0681	.0015
Self-pay	1.0 (360)	1.3 (667)	1.3 (45)	.0035	.2299
No charge	0.1 (31)	0.1 (68)	–^b^	.0792	.2873
Other	2.7 (995)	3.3 (1653)	3.6 (124)	.0020	.0828
Missing	0.2 (58)	0.1 (31)	–^b^	.0163	.1472
Patient location, % (n)					
Central counties (≥1 million pop)	16.8 (6192)	21.6 (10 956)	22.1 (769)	<.0001	<.0001
Fringe counties (≥1 million pop)	26.5 (9806)	26.5 (13 424)	25.9 (902)	.9556	.6401
Metro 250 000-999 000	23.0 (8509)	22.1 (11 201)	19.6 (684)	.1313	.0071
Metro 50 000-249 000	12.4 (4592)	10.4 (5273)	12.3 (429)	<.0001	.9064
Micropolitan	11.7 (4309)	10.1 (5125)	10.2 (356)	<.0001	.0989
Nonmetro/micropolitan	9.5 (3498)	9.1 (4593)	9.8 (341)	.3224	.7191
Missing	0.1 (42)	0.2 (94)	–^b^	.0451	.8884
Median household income percentile (ZIP code), % (n)					
0-25th	24.6 (9077)	25.8 (13 063)	27.3 (951)	.0413	.0342
26th-50th	29.3 (10 834)	29.3 (14 848)	28.3 (988)	.9729	.4710
51st-75th	25.7 (9489)	25.6 (12 994)	26.6 (925)	.9433	.4733
76th-100th	19.2 (7085)	17.9 (9084)	16.6 (577)	.0103	.0114
Unknown/missing	1.3 (463)	1.3 (678)	1.3 (44)	.4554	.9292

Abbreviations: AKI, acute kidney injury; CABG, coronary artery bypass graft; CKD, chronic kidney disease; CPB, cardiopulmonary bypass; pop, population; SE, standard error.

^a^AKI requiring dialysis is a subpopulation of the AKI group.

^b^< 30 patients.

**Table 2.** Clinical Characteristics and Outcomes at the Index Hospitalization and Readmission After the Index Hospitalization in Adults with CKD 3/4 Who Underwent Multivessel CABG, Valve Replacement/Repair, or CABG and Valve Replacement/Repair with CPB

**Characteristic**	**No AKI (N = 36 948)**	**AKI (N = 50 667)**	**AKI Requiring Dialysis^a^ (N = 3485)**	***P* Value (No AKI vs AKI)**	***P* Value (No AKI vs AKI Requiring Dialysis)**
**Clinical characteristics and outcomes at index hospitalization^b^**					
Common Charlson comorbidities during index hospitalization^c^, % (n)					
Congestive heart failure	45.1 (16 660)	63.0 (31 895)	77.7 (2707)	<.0001	<.0001
Diabetes with complications	50.2 (18 548)	58.3 (29 533)	60.7 (2116)	<.0001	<.0001
Myocardial infarction	34.2 (12 633)	44.9 (22 738)	45.9 (1599)	<.0001	<.0001
Diabetes without chronic complications	27.3 (10 105)	35.6 (18 042)	36.8 (1284)	<.0001	<.0001
Chronic pulmonary disease	24.1 (8889)	26.1 (13 229)	28.4 (990)	<.0001	.0002
Peripheral vascular disease	20.9 (7723)	21.4 (10 853)	22.5 (783)	.2432	.1572
Cerebrovascular disease	11.1 (4095)	12.7 (6441)	13.5 (470)	<.0001	.0037
CCI score					
Mean (SE)	3.9 (0.0)	4.9 (0.0)	6.1 (0.1)		
Median	4	4	5		
Range (Q1, Q3)	2, 4	3, 5	3, 8		
Additional clinical characteristics and procedures of interest^ e^, % (n)					
Cardiogenic shock	3.8 (1389)	11.8 (5993)	29.5 (1027)	<.0001	<.0001
Hypervolemia	10.7 (3943)	10.5 (5310)	10.5 (367)	.7373	.8966
Intra-aortic balloon pump	4.0 (1485)	9.1 (4601)	19.1 (667)	<.0001	<.0001
Received dialysis during hospitalization, % (n)	–^f^	6.9 (3485)	100.0 (3485)	<.0001	–^d^
Use of mechanical ventilation, % (n)					
24-96 consecutive hours	1.6 (594)	6.2 (3146)	18.9 (659)	<.0001	<.0001
>96 consecutive hours	–^g^	5.7 (2866)	27.7 (966)	<.0001	<.0001
Admission type, % (n)					
Nonelective admission	34.3 (12 662)	55.0 (27 871)	59.2 (2062)	<.0001	<.0001
Elective admission	65.6 (24 227)	44.8 (22 720)	40.6 (1415)	<.0001	<.0001
Clinical outcomes of interest, % (n)					
Postoperative cardiogenic shock	1.4 (509)	3.8 (1910)	9.1 (316)	<.0001	<.0001
Failure to rescue	–^h^	3.0 (1495)	19.9 (695)	<.0001	<.0001
Surgical site infection	–^i^	1.0 (494)	2.9 (100)	<.0001	<.0001
Disposition at discharge of index hospitalization, % (n)					
Routine	32.3 (11 941)	22.9 (11 594)	11.0 (385)	<.0001	<.0001
Transfer other (eg, skilled nursing facility, intermediate-care facility, another type of facility)	20.9 (7723)	30.0 (15 185)	36.1 (1259)	<.0001	<.0001
Home healthcare	45.1 (16 662)	41.3 (20 927)	24.3 (846)	<.0001	<.0001
Died in hospital	1.1 (404)	4.9 (2496)	26.5 (923)	<.0001	<.0001
Other^ j^	–^j^	–^j^	–^j^	.0120	.0103
**Readmission after index hospitalization**					
Total N	36 948	50 667	3485		
Had any readmission within 180 days	7568	14 428	1071	<.0001	<.0001
No. of readmissions within 180 days	1.4 (0)	1.5 (0)	1.6 (0)	<.0001	<.0001
Median (Q1, Q3)	1.0 (1, 1)	1.0 (1, 1)	1.0 (1, 2)	<.0001	<.0001
Mean (SE)	1.4 (0)	1.5 (0)	1.6 (0)	<.0001	<.0001
Time from index hospitalization discharge to first readmission within 180 days, % (n)					
0-15 days	40.0 (3025)	41.7 (6010)	40.1 (429)	.1122	.9668
16-30 days	17.0 (1286)	18.6 (2679)	21.4 (230)	.0491	.0166
31-60 days	17.2 (1300)	16.8 (2429)	20.2 (216)	.6752	.1243
61-90 days	10.3 (781)	8.8 (1271)	8.2 (88)	.0162	.1787
91-180 days	15.5 (1175)	14.1 (2039)	10.1 (108)	.0669	.0010
Median (Q1, Q3) LOS of first re-admission (days)	4.0 (2, 6)	4.0 (2, 7)	5.0 (2, 10)	<.0001	<.0001
Common primary diagnoses for first readmission, % (n)					
I13: Hypertensive heart disease and CKD	12.5 (942)	18.3 (2639)	21.6 (231)	<.0001	<.0001
T81: Complications of procedures, not elsewhere classified	6.7 (507)	6.7 (968)	8.7 (93)	.9850	.1008
A41: Other sepsis	5.5 (414)	6.3 (911)	8.7 (93)	.1035	.0033
N17: Acute kidney failure	3.4 (256)	4.2 (605)	5.1 (55)	<.0001	<.0001
I48: Atrial fibrillation and flutter	5.2 (394)	3.5 (498)	–^k^	.0438	.0665
Primary or secondary diagnosis of AKI during subsequent hospitalization, % (n)	41.8 (3165)	58.0 (8368)	67.4 (722)	<.0001	<.0001
Died during subsequent hospitalization, % (n)	4.1 (312)	5.7 (827)	9.4 (101)	.0005	<.0001

Abbreviations: AKI, acute kidney injury; CABG, coronary artery bypass graft; CCI, Charlson Comorbidity Index; CKD, chronic kidney disease; CPB, cardiopulmonary bypass; ECMO, extracorporeal membrane oxygenation; LOS, length of stay; SE, standard error.

^a^AKI requiring dialysis is a subpopulation of the AKI group.

^b^Index hospitalization includes time after surgery.

^c^See **Supplementary Table S5**.

^d^Chi-square test could be computed because at least 1 table cell has zero frequency (ie, all records in AKI requiring dialysis cohort have value of 1 for dialysis so there are no patients in this cohort with a value of 0 for dialysis).

Fewer than 1% of patients:

^e^Had other clinical characteristics/procedures of interest, including impella, ECMO, or surgical site infection.

^f^Received dialysis during index hospitalizations.

^g^Spent >96 consecutive hours on mechanical ventilation;

^h^With no AKI had failure to rescue.

^i^With no AKI had a surgical site infection.

^j^Were discharged to another destination (eg, discharged/transferred to short-term hospital or court/law enforcement; against medical advice; alive; destination unknown; unknown/missing).

^k^<30 patients.

Analyses were conducted using SAS Version 9.4 or later, with descriptive statistics (see **Supplementary Methods**) and incremental burden of AKI calculated and reported. Statistical comparisons between AKI and no AKI cohorts were conducted using Rao-Scott chi-square tests for congestive heart failure, myocardial infarction, cardiogenic shock, and dialysis status. Survey-weighted linear regression models for Charlson Comorbidity Index (CCI) scores, and *P* values are reported in-text. Significance was assumed at *P*< .05, and all statistical comparisons were performed against the no AKI cohort. The incremental burden associated with AKI with/without dialysis among patients with CKD 3/4 who underwent CPB was calculated as the difference in study measures between cohorts.

Multivariable regression analyses were performed to assess the effect of AKI on LOS, costs, and death during index hospitalization, as well as on readmissions. Generalized linear models with a gamma distribution and log-link function were used for LOS and costs, logistic regression for mortality, and Cox proportional hazards modeling for the first observed readmission within 180 days. For Cox proportional hazards models, we applied the SAS NMCAR option (analyzing all nonmissing data without dropping cases). For other multivariable models, 575 of 87 615 patients (~0.7%) were excluded due to missing values. No formal collinearity checks or additional regression diagnostics were performed for these models. Cardiogenic shock was considered as a covariate in multivariable models, however was ultimately excluded, as the timing of shock relative to AKI is unknown, and shock can occur as a postoperative complication. For demographic and clinical characteristic controls, see **Supplementary Methods**.

## RESULTS

### Characterizing Study Populations

**Figure 2.**
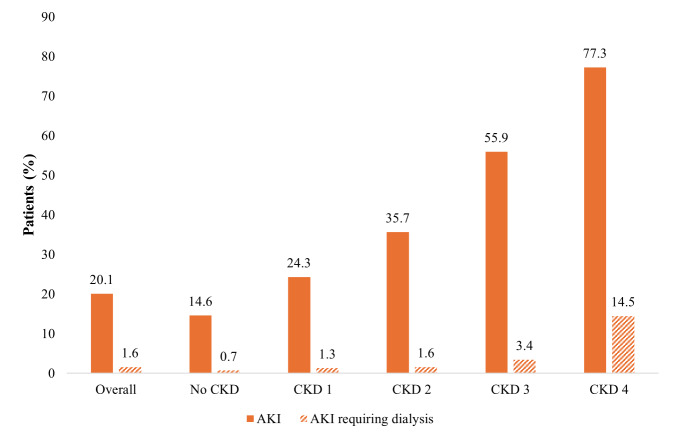
Incidence of AKI Within the Total Adult Cardiac Surgery Population^a^, by CKD Status Abbreviations: AKI, acute kidney injury; CABG, coronary artery bypass graft; CKD, chronic kidney disease; CPB, cardiopulmonary bypass. ^a^Adults who underwent multivessel CABG, valve replacement/repair, or combined CABG and valve replacement/repair with CPB.

**Incidence of AKI in total adult cardiac surgery population (incidence population):** In this population (N = 794 327; **Figure 1**), 20.1% of patients developed AKI during index hospitalization between 2016 and 2020. The incidence of AKI irrespective of dialysis requirement was comparable between males and females and increased over time from 17.4% (2016) to 21.9% (2020). The overall incidence of AKI requiring dialysis was 1.6% and increased over time from 1.1% (2016) to 1.9% (2020) (**Supplementary Table S2**). Irrespective of dialysis requirement, AKI incidence increased with CKD stage (**Figure 2**).

**Impact of CKD 3/4 in adult cardiac surgery population (analysis of incidence subpopulation) on outcomes:** This subpopulation comprised patients meeting additional criteria (N = 87 615; **Figure 1**). The outcomes in this subpopulation was analyzed as those who did or did not develop AKI during index hospitalization (no AKI: n = 36 948, 42.2%; AKI: n = 50 667, 57.8%). Of the patients with CKD 3/4 who developed AKI, 3485 (6.9%) also required dialysis (**Table 2**).

All subsequent results are reported from analysis of the adult CKD 3/4 cardiac surgery population (N = 87 615; **Figure 1**).

### Demographics and Hospital Characteristics: Adult CKD 3/4 Cardiac Surgery Population

**Table 1** reports the baseline characteristics and distributions of patients with CKD stages 3 or 4 who subsequently did or did not develop AKI after cardiac surgery. Mean age was significantly lower in the AKI group compared with the no-AKI group (no AKI, 70.5 years; AKI, 69.8 years [*P*< .0001]).

Differences were also observed with insurance payer type. Medicaid coverage was significantly higher in those with AKI compared with the no-AKI group (6.4% vs 4.3% [*P*< .0001]), although this did not differ significantly between no AKI and AKI requiring dialysis (73.6% vs 72.6% [*P* = .4351]) There was no significant difference in private insurance between no AKI and AKI groups, although rates of Medicare coverage were significantly lower in AKI requiring dialysis compared with no AKI ([*P* = .0015]) (**Table 1**).

While the geographic distribution was broadly similar between groups, a greater proportion of patients with AKI resided in central metropolitan counties compared with those without AKI (no AKI, 16.8%; AKI, 21.6% [*P*< .0001]) (**Table 1**). The distribution of surgery types across all patients in the CKD 3/4 cardiac surgery population regardless of AKI status was 59.1% multivessel CABG, 33.5% valve replacement/repair, and 14.5% combined CABG and valve replacement/repair (**Supplementary Table S3**). Demographics did not vary substantially by type of surgery. Rates of AKI were 59.3% for multivessel CABG, 57.1% for valve replacement/repair, and 61.6% for combined CABG and valve replacement/repair (**Supplementary Table S3**). Hospital characteristics were broadly consistent across AKI status or by surgery type, with similar distributions for teaching status, urban-rural designation, and bed size (**Supplementary Table S4**).

### Clinical Characteristics: Adult CKD 3/4 Cardiac Surgery Population

Several common comorbidities/diagnoses were observed at a higher rate in patients who developed AKI (including AKI requiring dialysis) compared with those who did not at index hospitalization, including congestive heart failure, myocardial infarction, cardiogenic shock, and dialysis (all [*P*< .0001]) (**Table 2**). Additional common comorbidity rates are shown in **Supplementary Table S5**.

Patients who developed AKI had higher CCI scores at index hospitalization (mean ± standard error [SE]: no AKI, 3.9 ± 0.0; AKI, 4.9 ± 0.0 [*P*< .0001], AKI requiring dialysis 6.1 ± 0.1 [*P*< .0001]). Elective admissions were more common in patients who did not develop AKI (65.6%) than in patients who developed AKI (44.8%) (**Table 2**).

### Outcomes of Index Hospitalization: Adult CKD 3/4 Cardiac Surgery Population

**Figure 3.**
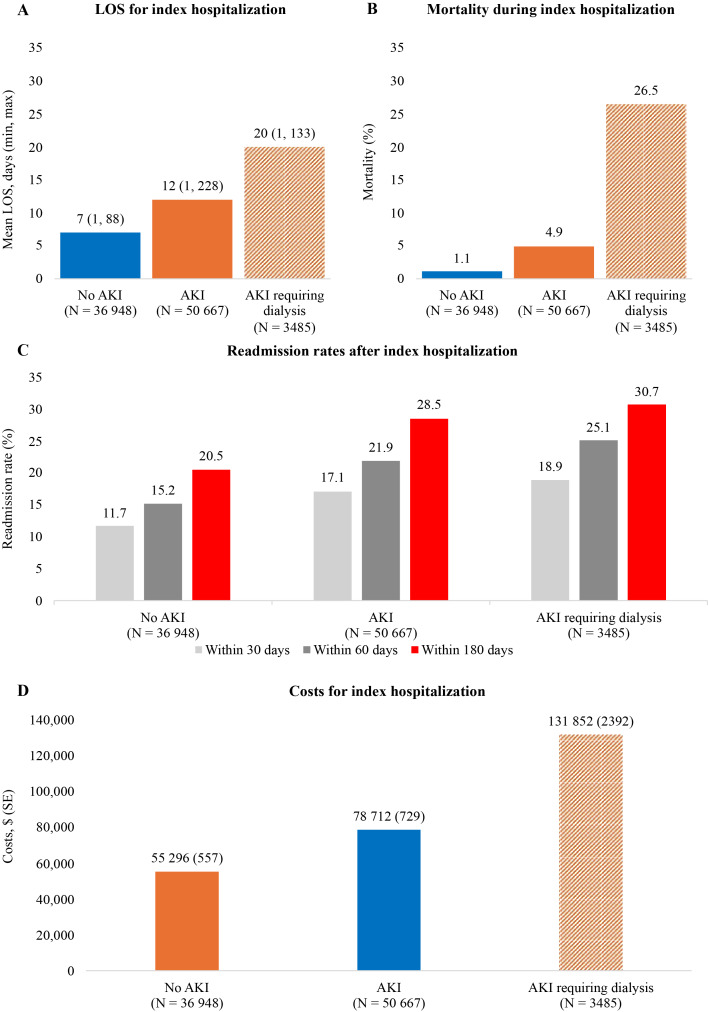
LOS for (**A**), Mortality During (**B**), Readmission Rates After (**C**) and Costs for (**D**) Index Hospitalization, within the Adult CKD 3/4 Cardiac Surgery Population,^a^ by AKI^b^ and Dialysis Status Abbreviations: AKI, acute kidney injury; CABG, coronary artery bypass graft; CKD, chronic kidney disease; CPB, cardiopulmonary bypass; LOS, length of stay; SE, standard error. ^a^Adults with a recorded diagnosis of CKD 3/4 who underwent multivessel CABG, valve replacement/repair, or CABG and valve replacement/repair with CPB. ^b^AKI requiring dialysis is a subpopulation of the AKI group.

The median LOS for index hospitalization was 5.0 days longer for patients who developed AKI vs patients with no AKI (12.0 vs 7.0 days), and 13.0 days longer in those with AKI requiring dialysis vs no AKI (20.0 vs 7.0 days; **Figure 3A**). In patients undergoing valve replacement/repair, those with AKI had a median LOS 6.0 days longer than those with no AKI (13.0 vs 7.0 days). For multivessel CABG, LOS was 4.0 days longer in patients with AKI (12.0 vs 8.0 days), and for combined CABG and valve replacement/repair LOS was 5.0 days longer (13.0 vs 8.0 days; **Supplementary Figure S1**).

Patients who developed AKI, and those who developed AKI requiring dialysis, had significantly higher rates of postoperative cardiogenic shock compared with patients with no AKI (no AKI, 1.4%; AKI, 3.8%; AKI requiring dialysis, 9.1% [*P* < .0001]) (**Table 2**).

During index hospitalization, significantly more patients who developed AKI, and who developed AKI requiring dialysis, spent 24-96 consecutive hours on mechanical ventilation compared with patients with no AKI (no AKI, 1.6%; AKI, 6.2% [*P* < .0001]; AKI requiring dialysis, 18.9% [*P* < .0001)]. Patients with no AKI were significantly more likely to have a routine discharge following index hospitalization (*P* < .0001), whereas significantly more patients who developed AKI, and who developed AKI requiring dialysis, were transferred to another facility at discharge (*P* < .0001) (**Table 2**).

### Mortality and Readmission: Adult CKD 3/4 Cardiac Surgery Population

Mortality during index hospitalization was significantly higher in patients with AKI, and in those who developed AKI requiring dialysis, compared with those with no AKI (no AKI, 1.1%; AKI, 4.9% [*P*  <  .0001]; AKI requiring dialysis, 26.5% [*P*  <  .0001]) (**Figure 3B**). Readmission within 30 days occurred in 11.7% of patients with no AKI, 17.1% of patients with AKI, and 18.9% of patients with AKI requiring dialysis. Within 180 days, readmission rates were 20.5% (no AKI), 28.5% (AKI), and 30.7% (AKI requiring dialysis; **Figure 3C**). Median LOS (Q1, Q3) for first readmission was 4.0 (2, 6) days for patients with no AKI, 4.0 (2, 7) days for those with AKI (*P* < .0001), and 5.0 (2, 10 [*P* < .0001]) days for those with AKI requiring dialysis. The most common reason (primary diagnosis) for first readmission was hypertensive heart and chronic kidney disease (no AKI, 12.5%; AKI, 18.3%; AKI requiring dialysis, 21.6%; *P* < .0001). Subsequent hospitalizations were associated with significantly higher rates of AKI diagnosis (no AKI, 41.8%; AKI, 58.0%; AKI requiring dialysis, 67.4%; [*P* < .0001]) (**Table 2**). Additional data on time from index discharge to first readmission is presented in **Supplementary Table S6**. In patients readmitted within 180 days after index hospitalization, mortality was significantly higher in those who developed AKI, and in those who developed AKI requiring dialysis, compared with those with no AKI (no AKI, 4.1%; AKI, 5.7%; [*P* = .0005]; AKI requiring dialysis, 9.4% [*P* < .0001]) (**Table 2**).

By type of cardiac surgery, mortality was highest among patients undergoing both CABG and valve replacement/repair (no AKI, 2.1%; AKI, 7.8%), whereas multivessel CABG alone had the lowest mortality rates during the index hospitalization (no AKI 0.9%; AKI, 4.2%) (**Supplementary Figure S2**).

### Cost of Index Hospitalization: Adult CKD 3/4 Cardiac Surgery Population

Average costs for index hospitalization were $23 416 higher for patients with AKI ($78 712) and $76 556 higher for patients with AKI requiring dialysis ($131 852), compared with patients with no AKI ($55 296; **Figure 3D**). Among patients with valve replacement/repair, those with AKI incurred $32 965 more in costs than those with no AKI ($96 325 vs $63 360). For multivessel CABG, patients with AKI incurred $19 875 more ($73 565 vs $53 690), and for combined CABG and valve replacement/repair, costs were $27 909 higher ($95 316 vs $67 407) compared with patients with no AKI (**Supplementary Figure S3**).

### Multivariable Analyses: Adult CKD 3/4 Cardiac Surgery Population

After controlling for demographic and clinical characteristics, an AKI diagnosis during index hospitalization in patients with CKD 3/4 was associated with an additional 3.98 inpatient days (*P* < .0001) and an extra $13 866 in costs (95% confidence interval [CI], $12 958-14 774 [*P* < .0001]). The presence of AKI was also associated with significantly higher odds of death during the index hospitalization (odds ratio 2.66, 95% CI, 2.25-3.14; *P* < .0001), and a higher risk of at least one readmission at 180 days (adjusted hazard ratio = 1.37, 95% CI, 1.308-1.432, *P* < .0001; **Table 3**).

**Table 3.** Multivariable Analyses

**Marginal Effect of AKI on LOS and Costs During Index Hospitalization**					
	**Marginal Effect**	**95% CI**		***P* Value**	
LOS (days)	3.98	3.82-4.14		<.0001	
Costs (USD)	$13 866	12 958−14 774		<.0001	
**Logistic Regression Analysis: Effect of AKI on Death During Index Hospitalization**					
	**Odds Ratio**	**95% CI**		***P* Value**	
No AKI	–^a^	–^a^		–^a^	
AKI	2.66	2.25-3.14		<.0001	
**Cox Regression Analysis: Effect of AKI on Readmissions**					
	***N* Hospitalizations**		**Multivariate Analysis**		
	**AKI**	**No AKI**	**Adjusted HR**	**95% CI**	***P* Value**
Readmission within 180 days			1.368	1.308-1.431	<.0001
Event	14 428	7568			
Censor	36 238	29 380			

Abbreviations: AKI, acute kidney injury; CI, confidence interval; HR, hazard ratio; LOS, length of stay; USD, US dollars.

^a^Reference values.

Note: All data presented are weighted estimates. Variables included as covariates are stated in the **Supplementary Methods**.

## DISCUSSION

This study evaluated the overall incidence of AKI by CKD stage in adults in the US undergoing cardiac surgery with CPB between 2016 and 2020, and showed that as the severity of CKD increased, incidence of AKI increased. Specifically, the adult CKD 3/4 cardiac surgery population (~11% of the total adult cardiac surgery population) made up 33% of AKI cases and ~50% of AKI cases requiring dialysis, suggesting this population is at higher risk in such settings–requiring heightened vigilance and closer monitoring.

While our findings broadly align with previous studies, to our knowledge this is one of the largest assessments of real-world data on the risk and impact (on both outcomes and costs) of AKI in patients with preexisting CKD undergoing cardiac surgery with CPB. Furthermore, we believe that the assessment of AKI in our study population of patients with CKD undergoing cardiac surgery with CPB addresses a heretofore underrepresented and possibly underrecognized group, vs previous studies.

In the adult CKD 3/4 cardiac surgery population, those who developed AKI had more comorbidities, longer hospital stays, greater healthcare costs, higher readmission rates through 180 days, and increased mortality, compared with patients with no AKI; patients who developed AKI requiring dialysis during index hospitalization had even worse outcomes than the overall AKI population. Logistic regression analysis indicated that patients who developed AKI were ~2.5 times more likely to die during index hospitalization than those with no AKI. A larger proportion of patients diagnosed with AKI died during subsequent readmissions, compared with the number who died during index hospitalization. Our findings augment previous investigations, showing perioperative AKI is independently associated with increases in short-term morbidity and healthcare costs, and long-term mortality, irrespective of the varying definitions of AKI, CKD, and surgery used across prior studies.^1–3,9^

As previously described, CKD is a known risk factor for AKI development, particularly in patients undergoing cardiac surgery, but it is often underdiagnosed.^19^ Prior studies reported that while ~25% of patients without CKD develop AKI following cardiac surgery, this rises to ~50% in patients with preexisting CKD.^10–12^ Indeed, data reported here concurs with similar studies that the incidence of postoperative AKI increases with worsening presurgical CKD stage.^20–22^ Preexisting CKD leads to a reduced renal functional reserve, hindering the ability of the kidney to compensate for subsequent injuries/insults, like AKI; the renal functional reserve may mask early, but clinically relevant, kidney injury.^23^ Considering the late presentation of elevated sCr, it is important for healthcare professionals to holistically evaluate kidney function and recognize that any prior kidney injury is associated with an increased risk of AKI which subsequently is a predictor of worse overall outcomes.

The development of AKI in patients undergoing cardiac surgery is also associated with increases in average per-patient costs. In patients with CKD 3/4 and an AKI diagnosis during index hospitalization, average costs were $23 416 more vs those with no AKI. This increase was even greater in patients who developed AKI requiring dialysis ($76 556 more vs no AKI). Across ~90 000 patients with CKD 3/4 undergoing cardiac surgery with CPB over a 5-year period, additional (unadjusted, aggregate) costs associated with an AKI diagnosis during index hospitalization exceeded $1.19 billion across the US. Multivariable analysis of the marginal effect of AKI in this study (controlling for factors such as surgery type, comorbidity burden, and demographics) indicated an additional $13 000 in costs and ~4 days’ additional LOS for patients who developed AKI, underscoring the importance of early identification and management.

We acknowledge several limitations of this study. Given the retrospective, descriptive, and observational design, all findings reflect associations rather than causal effects. No laboratory data were available to confirm CKD, ESKD, and AKI ICD-10-CM code-based diagnoses. Previous studies have found that AKI and CKD ICD-10-CM codes have high inpatient specificity but low sensitivity, consequently some cases of AKI and CKD may have been undercoded. Additionally, as our data derive from standard billing codes, some patients with CKD could have been overdiagnosed with AKI. This overcoding could conversely inflate the apparent incidence of AKI in CKD patients. Procedures were coded using ICD-10-PCS only and no information was available regarding concomitant medications, limiting considerations of comorbid drug toxicities. Readmissions were reported only for subsequent visits within the same state and year, potentially leading to underreporting. The database also lacks information on diagnoses and procedures that occurred outside the inpatient setting, so the full spectrum of patients’ comorbidities might not be captured. Moreover, important intraoperative variables such as packed red blood cell transfusion and CPB duration were not captured in the dataset, limiting the ability to fully assess contributors to AKI risk and outcomes. Limited information was available regarding long-term dialysis and receipt of dialysis during the admission and beyond.

Neither propensity score matching nor weighting were performed in this analysis, given the absence of a defined preoperative baseline period in the NRD and our descriptive study aim. We adjusted for baseline differences using standard multivariable regression, but residual confounding by indication may persist. As patients who developed AKI were more acutely ill (e.g. higher comorbidity burden, more emergency admissions), our observed outcome gaps could be partly exaggerated by underlying severity differences that are not fully captured. Furthermore, the timing of additional diagnoses during index hospitalization comparative to AKI was unavailable. Despite its limitations, this study highlights the increased incidence of AKI in patients with CKD undergoing either multivessel CABG, valve replacement/repair, or combined multivessel CABG and valve replacement/repair with CPB. Further, while many of the differences between groups in this analysis were numerically small, the extremely large sample size means our analyses were well powered to identify any significant differences between groups, lending credibility to our conclusions. This study demonstrates that AKI increases patient burden, negatively impacts clinical outcomes, and results in increased pressure and greater costs for healthcare systems. Importantly, we observed that although patients with CKD 3/4 represented a minority (11%) of all adults undergoing cardiac surgery with CPB, they accounted for one-third of all patients who went on to develop AKI, and half of all patients developing AKI requiring dialysis. The overrepresentation of patients with CKD 3/4 illustrates the substantial increase in AKI risk–and subsequent poorer outcomes, increased HCRU, and elevated costs–in this population.

## CONCLUSIONS

There is a clear need for targeted interventions to prevent and/or manage all forms of AKI, even mild injury, to help improve outcomes for all patients undergoing cardiac surgery with CPB. Future research should focus on prevention, alongside early detection and management, to further improve patient outcomes and decrease the burden that AKI in patients undergoing cardiac surgery with CPB places on patients and healthcare systems.

### Disclosures

N.P. has participated as a consultant or scientific advisory board member for Abbot Vascular and Alexion, AstraZeneca Rare Disease. R.C.A. has received honoraria from Edwards LifeSciences, Bioporto, and HLS Therapeutics Inc. In addition, he has participated as consultant or scientific advisory board member for Renibus Therapeutics Inc, and Alexion, AstraZeneca Rare Disease. T.J. was an employee of Alexion, AstraZeneca Rare Disease at the time of study conduction. C.S. and Y.W. are employees of Alexion, AstraZeneca Rare Disease, and own stock/options in Alexion, AstraZeneca Rare Disease. A.G. and J.M. are employees of RTI Health Solutions, which received compensation from Alexion for the design and conduct of the study. This study was funded by Alexion, AstraZeneca Rare Disease. Alexion, AstraZeneca Rare Disease was involved in the study design; in the collection, analysis, and interpretation of data; in the writing of the report; and in the decision to submit the article for publication.

## Supplementary Material

Online Supplementary Material

## Data Availability

Patient data used in this study were purchased from the Agency for Healthcare Research and Quality. The analysis plan was limited to the results highlighted in this manuscript. Additional information on accessing HCUP data is available here. Any queries or requests relating to the results presented in this manuscript should be directed to the corresponding author, Dr. Nicolas Pope (nicolas.pope%40musc.edu?subject=).

## References

[1] Xu J., Shen Bo, Fang Yi. (2015). Postoperative fluid overload is a useful predictor of the short-term outcome of renal replacement therapy for acute kidney injury after cardiac surgery. Medicine (Baltimore).

[2] Koc V., Delmas Benito L., de With E.. (2020). The effect of fluid overload on attributable morbidity after cardiac surgery: A retrospective study. Crit Care Res Pract.

[3] Alshaikh H. N., Katz N. M., Gani F.. (2018). Financial impact of acute kidney injury after cardiac operations in the United States. Ann Thorac Surg.

[4] Hsu R.K., Hsu C.Y. (2016). The role of acute kidney injury in chronic kidney disease. Semin Nephrol.

[5] Goyal A.. (2024). Acute Kidney Injury.

[6] Hu J., Chen R., Shaopeng L.. (2016). Global incidence and outcomes of adult patients with acute kidney injury after cardiac surgery: a systematic review and meta-analysis. J Cardiothorac Vasc Anesth.

[7] Mao H., Katz N., Ariyanon W.. (2014). Cardiac surgery-associated acute kidney injury. Blood Purif.

[8] Zarbock A., Weiss R., Albert F.. (2023). Epidemiology of surgery associated acute kidney injury (EPIS-AKI): A prospective international observational multi-center clinical study. Intensive Care Med.

[9] Meersch M., Schmidt C., Zarbock A. (2017). Perioperative acute kidney injury: an under-recognized problem. Anesth Analg.

[10] Hsu C. Y., Ordoñez J. D., Chertow G. M.. (2008). The risk of acute renal failure in patients with chronic kidney disease. Kidney Int.

[11] Wu V. C., Huang T.-M., Lai C.-F.. (2011). Acute-on-chronic kidney injury at hospital discharge is associated with long-term dialysis and mortality. Kidney Int.

[12] Zhang D., Teng D., Luo Z.. (2023). Risk factors and prognosis of acute kidney injury after cardiac surgery in patients with chronic kidney disease. Blood Purif.

[13] Husain-Syed F., Ferrari F., Sharma A.. (2019). Persistent decrease of renal functional reserve in patients after cardiac surgery-associated acute kidney injury despite clinical recovery. Nephrol Dial Transplant.

[14] Ostermann M., Kunst G., Baker E.. (2021). Cardiac surgery associated AKI prevention strategies and medical treatment for CSA-AKI. J Clin Med.

[15] Schurle A., Koyner J. L. (2021). CSA-AKI: incidence, epidemiology, clinical outcomes, and economic impact. J Clin Med.

[16] Machado M. N., Nakazone M. A., Maia L. N. (2014). Prognostic value of acute kidney injury after cardiac surgery according to kidney disease: improving global outcomes definition and staging (KDIGO) criteria. PLoS One.

[17] Ronco C., Rosner M. H. (2012). Acute kidney injury and residual renal function. Crit Care.

[18] Agency for Healthcare Research and Quality Introduction to the NRD.

[19] Centers for Medicare & Medicaid Services (2020). Chronic kidney disease often undiagnosed in Medicare beneficiaries.

[20] Shlipak M. G., Coca S. G., Wang Z.. (2011). Presurgical serum cystatin C and risk of acute kidney injury after cardiac surgery. Am J Kidney Dis.

[21] Fujii T., Uchino S., Takinami M.. (2014). Subacute kidney injury in hospitalized patients. Clin J Am Soc Nephrol.

[22] Su C. C., Chen J.-Y., Chen S.-Y.. (2023). Outcomes associated with acute kidney disease: a systematic review and meta-analysis. EClinicalMedicine.

[23] Sharma A., Mucino M. J., Ronco C. (2014). Renal functional reserve and renal recovery after acute kidney injury. Nephron Clin Pract.

