# Supplementary material for: Impact of Acute Kidney Injury on Hospitalization Outcomes in Patients with Chronic Kidney Disease Undergoing Cardiac Procedures with Cardiopulmonary Bypass in the United States
Source: J Health Econ Outcomes Res. 2026 Aug 14;13(2):77–86. doi: 10.36469/001c.163558 (PMC13477740; doi:10.36469/001c.163558)
Supplement: Supplementary file 1 [file jheor_2026_13_2_163558_359349.pdf]

Journal of Health Economics and Outcomes Research

Online Supplementary Material

Impact of Acute Kidney Injury on Hospitalization Outcomes in Patients with Chronic Kidney Disease Undergoing Cardiac Procedures with Cardiopulmonary Bypass in the United States. *JHEOR*. 2026;13(2):77-86. doi:10.36469/jheor.2026.163558

Supplementary Methods **S2**

Table S1A: Summary of ICD-10 Coding **S2**

Table S1B: Summary of ICD-10-CM Coding and CCI Scoring for Comorbidities **S3**

Table S2: Percentages of Patients with AKI and AKI with Dialysis: Total Adult Cardiac Surgery Population **S4**

Table S3: Demographic Characteristics at Index Hospitalization Within the Adult CKD 3/4 Cardiac Surgery Population, by Type of Cardiovascular Surgery and AKI Status **S5**

Table S4: Hospital Characteristics of Hospitalizations for CPB and Procedures of Interest within the Adult CKD 3/4 Cardiac Surgery Population by AKI and Dialysis Status **S6**

Table S5: Additional Clinical Characteristics at Index Hospitalization Within the Adult CKD 3/4 Cardiac Surgery Population,by AKI and Dialysis Status **S6**

Table S6: Additional Data: Time From Index Hospitalization Discharge to First Readmission within the Adult CKD 3/4 Cardiac Surgery Population,by AKI and Dialysis Status **S7**

Figure S1: Length of Stay for Index Hospitalization Within the Adult CKD 3/4 Cardiac Surgery Population,by Surgery Type **S7**

Figure S2: Mortality During Index Hospitalization Within the Adult CKD 3/4 Cardiac Surgery Population, by Surgery Type **S8**

Figure S3: Costs During the Index Hospitalization Within Adult CKD 3/4 Cardiac Surgery Population, by Surgery Type **S8**

This supplementary material has been provided by the authors to give readers additional information about their work.

This is an open-access article distributed under the terms of the Creative Commons Attribution 4.0 International License (CCBY-4.0). View this license's legal deed at http://creativecommons.org/licenses/by/4.0 and legal code at http://creativecommons.org/licenses/by/4.0/legalcode for more information.

SUPPLEMENTARY METHODS

Analysis years (2016-2020) aligned with the implementation of ICD-10-CM coding. An average of 29 states contributed data each year between 2016 and 2020. Unweighted, the NRD contains data from approximately 17 million discharges; when weighted, it represents about 32 million discharges.

Descriptive statistics included means and standard error (SE), or medians and ranges for continuous variables; and frequency distributions for categorical variables.

Multivariate analysis controlled for the following demographic and clinical characteristics: age, sex, insurance payer type, hospital urban-rural designation, hospital teaching status, CCI score, admission type (elective vs nonelective), mechanical ventilation, procedure type (multivessel CABG, valve replacement/repair, CABG and valve replacement/repair), and diagnosis of hypervolemia.

SUPPLEMENTARY TABLES AND FIGURES

Table S1A. Summary of ICD-10 Coding

Conditions	ICD-10 Code	Code Description
Stage I CKD	N18.1x	Chronic kidney disease, stage 1
Stage II CKD	N18.2x	Chronic kidney disease, stage 2
Stage III CKD	N18.3	Chronic kidney disease, stage 3
	N18.30	Chronic kidney disease, stage 3 unspecified (implemented in 2020)
	N18.31	Chronic kidney disease, stage 3a (implemented in 2020)
	N18.32	Chronic kidney disease, stage 3b (implemented in 2020)
Stage IV CKD	N18.4x	Chronic kidney disease, stage 4
Stage V CKD	N18.5x	Chronic kidney disease, stage 5
ESRD	N18.6x	End-stage renal disease
Severe renal disease (additional codes)	I12.0	Hypertensive chronic kidney disease with stage 5 CKD or ESRD
	I13.11	Hypertensive heart and CKD without heart failure with stage 5 CKD or ESRD
	I13.2	Hypertensive heart and CKD with heart failure and with stage 5 CKD or ESRD
Unspecified CKD	N18.9x	CKD, unspecified
Acute kidney injury	N17.xx	Acute kidney failure
	N99.0	Postprocedural (acute) (chronic) kidney failure
Multivessel CABG	0211	Bypass, coronary artery, 2 arteries
	0212	Bypass, coronary artery, 3 arteries
	0213	Bypass, coronary artery, ≥4 arteries
Single vessel CABG	0210	Bypass, coronary artery, 1 artery
Valve replacement or repair		
Repair	02QF	Repair, aortic valve
	02QG	Repair, mitral valve
	02QH	Repair, pulmonary valve
	02QJ	Repair, tricuspid valve
Replace	02RF	Replace, aortic valve
	02RG	Replace, mitral valve
	02RH	Replace, pulmonary valve
	02RJ	Replace, tricuspid valve
Coronary artery repair	02Q0	Repair, coronary artery, 1 artery
	02Q1	Repair, coronary artery, 2 arteries
	02Q2	Repair, coronary artery, 3 arteries
	02Q3	Repair, coronary artery, ≥4 arteries
Cardiopulmonary bypass	5A1221Z	Performance of cardiac output, continuous

Table S1A. Summary of ICD-10 Coding

Conditions	ICD-10 Code	Code Description
Dialysis procedures	5A1D00Z	Performance of urinary filtration, single
	5A1D60Z	Performance of urinary filtration, multiple
	5A1D70Z	Performance of urinary filtration, intermittent, <6 hours/day
	5A1D80Z	Performance of urinary filtration, prolonged intermittent, 6-18 hours/day
	5A1D90Z	Performance of urinary filtration, continuous, greater than 18 hours/day
	3E1M39Z	Irrigation of peritoneal cavity using dialysate, percutaneous approach
	Z99.2	Dependence on renal dialysis
Dialysis dependence	Z49.XX	Encounter for care involving renal dialysis
Other dialysis codes	0WHG33Z	Insertion of infusion device into peritoneal cavity, percutaneous approach
Kidney transplant	Z94.0x	Kidney transplant status
	0TY0	Transplantation kidney, right
	0TY1	Transplantation kidney, left
Mechanical ventilation	5A1935Z	Respiratory ventilation, <24 consecutive hours
	5A1945Z	Respiratory ventilation, 24-96 consecutive hours
	5A1955Z	Respiratory ventilation, >96 consecutive hours
Intra-aortic balloon pump	5A02210	Assistance with cardiac output using balloon pump, continuous
Impella	Z95.811	Presence of heart assist device
Cardiogenic shock	R57.0	Cardiogenic shock
Postoperative cardiogenic shock	T81.11XA	Postprocedural cardiogenic shock, initial encounter
Extracorporeal membrane oxygenation (ECMO)	Z92.81	Personal history of ECMO
Surgical site infection	T81.4	Infection following a procedure
Hypervolemia	E87.7	Fluid overload
Failure to rescue (death and one of the conditions below)		
Stroke	I63	Cerebral infarction
	I64	Stroke not specified as hemorrhage or infarction
Prolonged mechanical ventilation	5A1945Z	Respiratory ventilation, 24-96 consecutive hours
	5A1955Z	Respiratory ventilation, >96 consecutive hours

Table S1B. Summary of ICD-10-CM Coding and CCI Scoring for Comorbidities

Comorbidities	CCI Score	ICD-10-CM Codes
Myocardial infarction	1	I21, I22, I252
Congestive heart failure	1	I110, I130, I132, I255, I420, I425, I426, I427, I428, I429, P290, I43, I50
Peripheral vascular disease	1	I70, I71, I731, I738, I739, I771, I790, I791, I798, K551, K558, K559, Z958, Z959
Cerebrovascular disease	1	G45, G46, I60, I61, I62, I63, I64, I65, I66, I67, I68, H340, H341, H342
Dementia	1	F01, F02, F03, F04, F05, G30, G94, R54, F061, F068, G132, G138, G310, G311, G312, G914, R4181
Chronic pulmonary disease	1	J40, J41, J42, J43, J44, J45, J46, J47, J60, J61, J62, J63, J64, J65, J66, J67, J684, J701, J703
Rheumatic disease	1	M05, M32, M33, M34, M06, M315, M351, M353, M360
Peptic ulcer disease	1	K25, K26, K27, K28
Liver disease, mild	1	B18, K73, K74, K700, K701, K702, K703, K709, K717, K713, K714, K715, K760, K762, K763, K764, K768, K769, Z944
Diabetes without chronic complications	1	Main codes: E08, E09, E10, E11, E13 + relevant subcodes: E**.0x, E**.1x, E**.6x, E**.8x, E**.9x
Renal disease, mild to moderate	1	I129, I130, I1310, N03, N05, N181, N182, N183, N184, N189, Z940
Diabetes with complications	2	Main codes: E08, E09, E10, E11, E13 + relevant subcodes: E**.2x, E**.3x, E**.4x, E**.5x

Hemiplegia or paraplegia	2	G041, G114, G800, G801, G802, G81, G82, G83
Any malignancy	2	C0, C1, C2, C30, C31, C32, C33, C34, C37, C38, C39, C40, C41, C43, C45, C46, C47, C48, C49, C50, C51, C52, C53, C54, C55, C56, C57, C58, C60, C61, C62, C63, C76, C801, C81, C82, C83, C84, C85, C88, C9
Liver disease, moderate-severe	3	K704, K711, K721, K729, K765, K766, K767, I850, I864
Renal disease, severe	3	I120, I1311, I132, N185, N186, N19, N250, Z49, Z992
HIV infection, no AIDS	3	B20
Metastatic solid tumor	6	C77, C78, C79, C800, C802
AIDS	6	B37, C53, B38, B45, A072, B25, G934, B00, B39, A073, C46, C81, C82, C83, C84, C85, C86, C87, C88, C89, C90, C91, C92, C93, C94, C95, C96, A31, A15, A16, A17, A18, A19, B59, Z8701, A812, A021, B58, R64

Abbreviations: CABG, coronary artery bypass graft; CCI, Charlson Comorbidity Index; CKD, chronic kidney disease; ECMO, extracorporeal membrane oxygenation; ESRD, end-stage renal disease; ICD-10(-CM), *International Classification of Diseases, Tenth Revision (Clinical Modification).*

Table S2. Percentages of Patients with AKI and AKI with Dialysis: Total Adult Cardiac Surgery Population (N = 794 327)[a]

Population	Patients with AKI Among Patients with CPB and Surgeries of Interest (n = 159 952), %	Patients with AKI Requiring Dialysis Among Patients with CPB and Surgeries of Interest (n = 12 703), %
All hospitalizations	20.1	1.6
Age group, years		
18-34	20.6	2.3
35-44	18.2	2.2
45-54	15.5	1.4
55-64	16.5	1.3
65-74	20.4	1.5
75-84	26.6	2.1
≥85	32.7	2.5
Sex		
Male	20.4	1.5
Female	19.6	1.9
Year of hospitalization		
2016	17.4	1.1
2017	18.0	1.3
2018	20.7	1.7
2019	21.2	1.8
2020	21.9	1.9

Abbreviations: AKI, acute kidney injury; CABG, coronary artery bypass graft; CPB, cardiopulmonary bypass.
[a]Adults who underwent multivessel CABG, valve replacement/repair, or combined CABG and valve replacement/repair with CPB.

Table S3. Demographic Characteristics at Index Hospitalization Within the Adult CKD 3/4 Cardiac Surgery Population,[a] by Type of Cardiovascular Surgery and AKI Status

**Characteristic**	**Overall (N = 87 615)**		**Multivessel CABG (n = 51 773; 59.1%)**		**Valve Replacement/ Repair (n = 29 372; 33.5%)**		**CABG and Valve Replacement/ Repair (n = 12 699; 14.5%)**	
	**No AKI**	**AKI**	**No AKI**	**AKI**	**No AKI**	**AKI**	**No AKI**	**AKI**
Total N (%)	36 948 (42.2)	50 667 (57.8)	21 070 (40.7)	30 703 (59.3)	12 610 (42.9)	16 762 (57.1)	4877 (38.4)	7822 (61.6)
Mean (SE) age, years	70.5 (0.1)	69.8 (0.1)	70.5 (0.1)	69.9 (0.1)	71.4 (0.1)	70.3 (0.1)	73.3 (0.2)	72.5 (0.2)
Sex, %								
Male	71.0	72.9	76.9	76.5	63.5	67.5	74.1	73.7
Female	29.0	27.1	23.1	23.5	36.5	32.5	25.9	26.3
Insurance payer type, %								
Medicare	73.6	71.4	73.5	70.8	76.2	74.5	81.8	79.8
Medicaid	4.3	6.4	4.2	6.4	4.0	6.0	2.5	3.9
Private	18.2	17.4	18.0	17.7	17.0	15.4	13.1	12.0
Other/missing	4.0	4.8	4.2	5.1	2.9	4.1	2.6	4.3
Patient location, %								
Central counties of metro areas of ≥1 million population	16.8	21.6	16.0	21.1	18.4	22.5	16.1	21.1
Fringe counties of metro areas of ≥1 million population	26.5	26.5	26.5	26.2	26.6	27.0	26.4	27.4
Counties in metro areas of 250 000-999 999 population	23.0	22.1	23.2	22.4	22.4	21.7	22.3	22.2
Counties in metro areas of 50 000-249 999 population	12.4	10.4	12.3	10.4	13.1	10.7	13.2	11.1
Micropolitan counties	11.7	10.1	12.2	10.5	10.6	9.3	11.8	9.1
Not metropolitan or micropolitan counties/ missing	9.6	9.3	9.8	9.3	9.0	8.7	10.2	9.1
Median household income for patient's zip code %								
0-25th percentile	24.6	25.8	25.3	26.4	21.8	24.0	21.4	22.9
26th-50th percentile (median)	29.3	29.3	29.9	29.7	29.6	28.6	31.7	29.2
51st-75th percentile	25.7	25.6	25.0	25.6	26.6	25.9	26.2	26.6
76th-100th percentile	19.2	17.9	18.6	17.0	20.5	20.2	19.2	20.0
Unknown/missing	1.3	1.3	1.2	1.4	1.4	1.3	1.5	1.3

Abbreviations: AKI, acute kidney injury; CABG, coronary artery bypass graft; CPB, cardiopulmonary bypass.
[a]Patients (≥18 years) with a recorded diagnosis of stage 3/4 CKD who underwent multivessel CABG, valve replacement/repair, or CABG and valve replacement/ repair with CPB.

Table S4. Hospital Characteristics of Hospitalizations for CPB and Procedures of Interest Within the Adult CKD 3/4 Cardiac Surgery Population[a] by AKI and Dialysis Status

**Characteristic**	**Overall, %**		**Multivessel CABG, %**		**Valve Replacement/ Repair, %**		**CABG and Valve Replacement/Repair, %**	
	**No AKI (N = 36 948)**	**AKI (N = 50 667)**	**No AKI (N = 21 070)**	**AKI (N = 30 703)**	**No AKI (N = 12 610)**	**AKI (N = 16 762)**	**No AKI (N = 4877)**	**AKI (N = 7822)**
Teaching status of hospital								
Metropolitan nonteaching	13.0	13.7	14.6	15.0	10.7	11.8	13.2	13.5
Metropolitan teaching	84.5	84.5	82.6	82.9	87.6	87.0	85.1	85.0
Nonmetropolitan hospital	2.4	1.8	2.8	2.2	1.6	1.2	1.7	1.5
Hospital urban-rural designation								
Large metropolitan areas with ≥1 million residents	52.4	57.4	49.8	55.3	57.4	61.4	54.7	58.4
Small metropolitan areas with <1 million residents	45.2	40.8	47.4	42.6	40.9	37.4	43.6	40.1
Micropolitan areas	2.3	1.7	2.6	2.1	1.5	1.1	1.4	1.4
Not metropolitan or micropolitan (nonurban residual)	0.2	0.1	0.2	0.1	–[b]	–[b]	–[b]	–[b]
Hospital bed size								
Small	10.4	10.0	10.8	10.4	9.3	9.5	10.7	10.4
Medium	23.3	23.0	24.4	24.0	22.6	21.6	24.0	22.5
Large	66.3	67.0	64.8	65.6	68.1	68.9	65.3	67.0

Abbreviations: AKI, acute kidney injury; CABG, coronary artery bypass graft; CKD, chronic kidney disease; CPB, cardiopulmonary bypass.
[a]Patients (≥18 years) with a recorded diagnosis of stage 3/4 CKD who underwent multivessel CABG, valve replacement/repair, or CABG and valve replacement/repair with CPB.
[b]<30 patients.

Table S5. Additional Clinical Characteristics at Index Hospitalization[a] Within the Adult CKD 3/4 Cardiac Surgery Population,[b] by AKI and Dialysis Status

**Characteristic**	**No AKI**	**AKI**	**AKI requiring dialysis[c]**
Total N	36 948	50 667	3485
Common Charlson comorbidities during index hospitalization, %[d]			
AIDS	5.2	11.5	26.4
Dementia	4.3	6.3	8.6
Liver disease, mild	2.8	3.2	5.0
Rheumatic disease	3.3	2.8	2.8
Any malignancy	2.6	2.8	3.4

Abbreviations: AKI, acute kidney injury; CABG, coronary artery bypass graft; CKD, chronic kidney disease; CPB, cardiopulmonary bypass.
[a]Index hospitalization includes time after operation.
[b]Patients (≥18 years) with a recorded diagnosis of stage 3/4 CKD who underwent multivessel CABG, valve replacement/repair, or CABG and valve replacement/repair with CPB.
[c]AKI requiring dialysis is a subpopulation of the AKI group.
[d]Comorbidities present in <2% of the population included hemiplegia or paraplegia, peptic ulcer disease, moderate to severe liver disease, HIV infection, and metastatic solid tumor.

Table S6. Additional Data: Time From Index Hospitalization Discharge to First Readmission Within the Adult CKD 3/4 Cardiac Surgery Population,[a] by AKI and Dialysis Status

Characteristic	No AKI	AKI	AKI Requiring Dialysis[b]
Total N	36 948	50 667	3485
Time from index hospitalization discharge to first readmission within 180 days, %[c]			
16-30 days	17.0	18.6	21.4
31-60 days	17.2	16.8	20.2
61-90 days	10.3	8.8	8.2

Abbreviations: AKI, acute kidney injury; CABG, coronary artery bypass graft; CKD, chronic kidney disease; CPB, cardiopulmonary bypass.
[a]Patients (≥18 years) with a recorded diagnosis of stage 3/4 CKD who underwent multivessel CABG, valve replacement/repair, or CABG and valve replacement/repair with CPB.
[b]AKI requiring dialysis is a subpopulation of the AKI group.
[c]Among patients with at least 1 readmission within 180 days.

Figure S1. LOS for Index Hospitalization Within the Adult CKD 3/4 Cardiac Surgery Population,[a] by Surgery Type

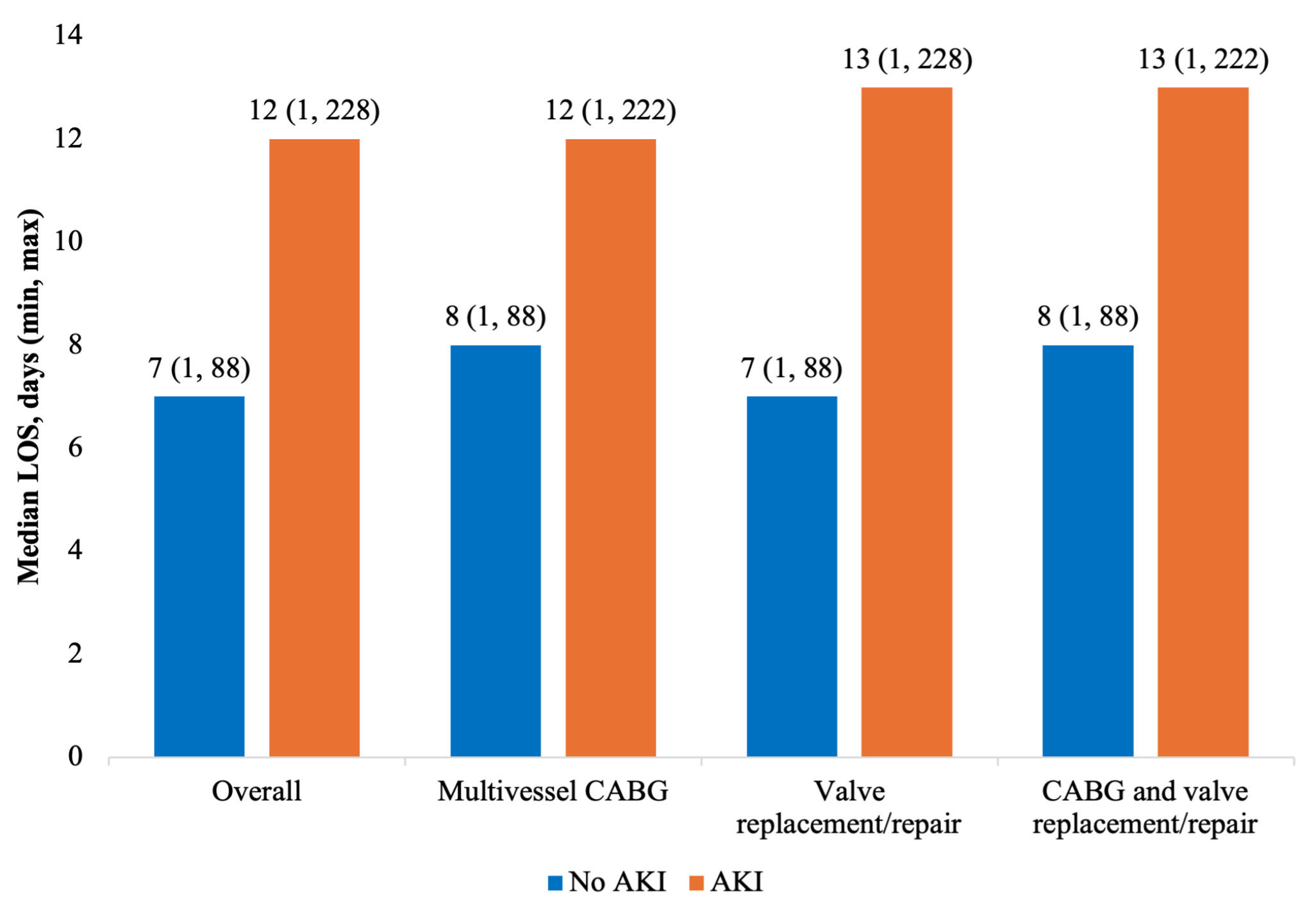

Abbreviations: AKI, acute kidney injury; CABG, coronary artery bypass graft; CKD, chronic kidney disease; CPB, cardiopulmonary bypass; LOS, length of stay.
[a]Patients (≥18 years) with a recorded diagnosis of stage 3/4 CKD who underwent multivessel CABG, valve replacement/repair, or CABG and valve replacement/repair with CPB.

Figure S2. Mortality During the Index Hospitalization Within the Adult CKD 3/4 Cardiac Surgery Population,[a] by Surgery Type

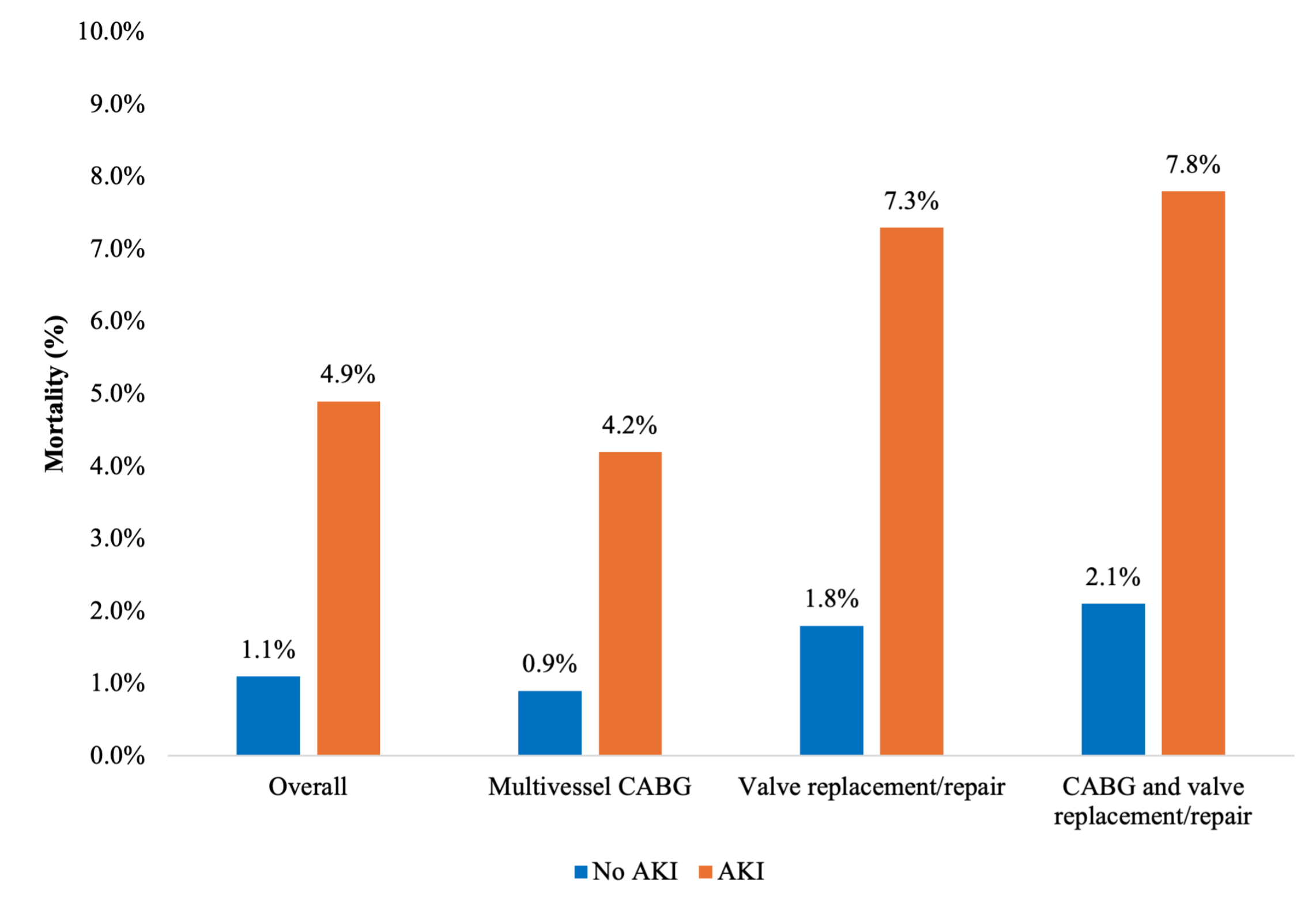

Abbreviations: AKI, acute kidney injury; CABG, coronary artery bypass graft; CKD, chronic kidney disease; CPB, cardiopulmonary bypass.
[a]Patients (≥18 years) with a recorded diagnosis of stage 3/4 CKD who underwent multivessel CABG, valve replacement/repair, or CABG and valve replacement/repair with CPB.

Figure S3. Costs During the Index Hospitalization Within Adult CKD 3/4 Cardiac Surgery Population,[a] by Surgery Type

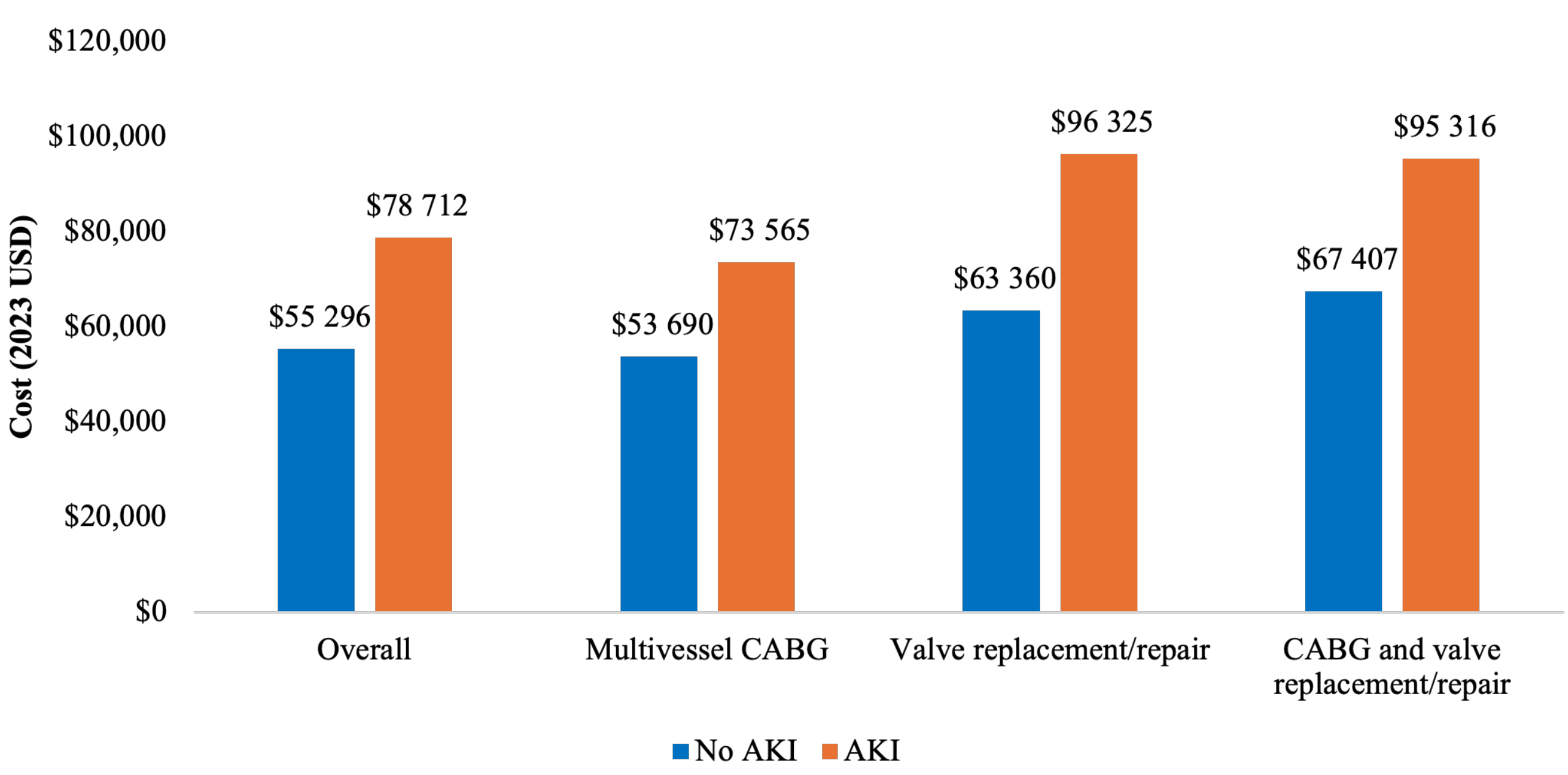

Abbreviations: CABG, coronary artery bypass graft; CKD, chronic kidney disease; CPB, cardiopulmonary bypass; USD, US dollars.
[a]Patients (≥18 years) with a recorded diagnosis of stage 3/4 CKD who underwent multivessel CABG, valve replacement/repair, or CABG and valve replacement/repair with CPB.
